# A Perspective on the Applications of Triphasic Gas Storage in Electrochemical Systems

**DOI:** 10.1002/advs.202514182

**Published:** 2025-10-30

**Authors:** Zhongkai Li, Corin T. Scott, Taku Suzuki‐Osborne, John P. Lowe, Dominic Taylor, Mariolino Carta, Neil B. McKeown, Andrew D. Burrows, Lucia H. Mascaro, Frank Marken

**Affiliations:** ^1^ Department of Chemistry University of Bath Claverton Down BA2 7AY UK; ^2^ Chemical Characterisation Facility University of Bath Bath BA2 7AY UK; ^3^ EaStCHEM School of Chemistry University of Edinburgh Joseph Black Building, David Brewster Road Edinburgh Scotland EH9 3JF UK; ^4^ Faculty of Science and Engineering Department of Chemistry College of Science Swansea University Grove Building, Singleton Park Swansea SA2 8PP UK; ^5^ Department of Chemistry Federal University of São Carlos São Carlos SP 13565–905 Brazil

**Keywords:** energy storage, gas adsorption, gas tunnel, nitrogen reduction, oxygen reduction

## Abstract

Microporous materials store gases under dry conditions (e.g., hydrogen or oxygen via physisorption), but in some cases microporous materials also show triphasic (e.g., in a solid|gas|liquid system) gas storage in the presence of humidity/water. This is exploited recently to enhance gas solubility in aqueous media (in microporous deposits or in “microporous water”) aided by microporous materials. Data obtained from NMR spectroscopy shows stored H_2_ within particles of a polymer of intrinsic microporosity (PIM‐1) suspended in water, which supports the concept and conclusions of triphasic gas storage derived from accelerated electrochemical reactions. This can be important for accelerating both electrocatalytic gas evolution as well as gas‐consuming electrocatalytic processes (e.g., in O_2_ to H_2_O_2_ or N_2_ to NH_3_ conversions). Comparison can be made between this observed acceleration in electrocatalysis and enzyme‐catalytic processes in nature, where enzymes are equipped with “gas tunnel” transport, for example, for producing ammonia in nitrogenases. This perspective examines this analogy and focuses primarily on the use of i) metal–organic frameworks (MOFs) and ii) polymers of intrinsic microporosity (PIMs). Gas binding under wet and dry conditions is contrasted. Reactions involving oxygen reduction, nitrogen reduction, hydrogen evolution/oxidation, and related applications in triphasic energy storage are discussed.

## Graphical abstract

Gas storage in microporous materials positioned locally at an electrode or electrocatalyst surface enhances electrochemical processes.

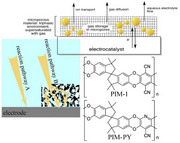

## 1 Introduction to Triphasic Processes and Interfaces

In the current phase of global energy transition, sustainable hydrogen gas provides a powerful and clean energy carrier alternative to traditional fossil fuels, inspiring the increase in recent research activity devoted to the storage and utilisation of hydrogen gas.^[^ ^1^ ^]^ In the energy storage sector, water‐based rechargeable aqueous metal‐air or metal‐gas batteries (electrochemical cells generally composed of an anode, gas‐breathing cathode, ion‐conducting membrane, and electrolyte)^[^ ^2^ ^]^ have been proposed with metals such as zinc, providing effective anode technology. In nickel‐hydrogen battery technology used in premillennium space technology, the anode is based on pressurised hydrogen gas and offers high durability and performance in aqueous KOH.^[^ ^3^ ^]^ The corresponding concept of rechargeable “hydrogen/oxygen batteries” in aqueous environments has been investigated, but is hampered by the problem of poor solubility (i.e., high pressure) of gaseous reactants in aqueous media/electrolyte.^[^ ^4^, ^5^ ^]^ When working with poorly soluble gaseous reactants, diffusional fluxes are low and bubble growth at the electrode surface can lead to the blocking of surface active sites and increased resistivity.^[^ ^6^ ^]^ In some cases, usually under higher pressure, storage by gas hydrates can be beneficial^[^ ^7^, ^8^ ^]^ and these can form in hydrophobic pores.^[^ ^9^ ^]^ For example, methane storage in wet porous carbon may occur via clathrate formation.^[^ ^10^ ^]^ Linked to these cases of gas storage and reactivity, it has recently been shown that a triphasic environment based on microporous materials (interacting with both liquid an gas) can help with i) reducing surface blocking by gas bubbles at electrode/catalyst surfaces and ii) increasing the apparent concentration (flux) of a gas locally at the electrode, which enhances reactivity and possibly alters reaction pathways.^[^ ^11^, ^12^ ^]^

Mason and coworkers developed the closely related concept of “microporous water” with dispersed ZIF‐8 or ZIF‐67 metal–organic frameworks enhancing gas storage.^[^ ^13^ ^]^ To date, there is an ever‐expanding range of microporous materials invented for storing/separating gases by adsorption.^[^ ^14^ ^]^ Pores with a diameter of <2 nm are referred to as micropores, which form interlinked networks within solids that impart unique properties to microporous materials, such as a high surface to mass or volume ratio.^[^ ^15^, ^16^ ^]^ The large surface area provided by internal micropores creates ample space for transport or storage of gases, such as hydrogen.

Microporous materials deposited at an electrode surface (i.e., a gas management layer) store gas as adsorbent and/or as nanobubbles, increasing gaseous solute flux to the electrode.^[^ ^17^ ^]^ Therefore, the creation of a triphasic environment (gaseous nanobubble, aqueous electrolyte, solid microporous material) increases the apparent concentration of gases at the electrode, effectively increasing gas solubility, changing reactivities and potentially mechanisms, while also preventing the formation of nanobubbles directly on the catalyst/electrode, which may block active sites and inhibit reactivity. Research on the use of these microporous materials in aqueous electrolyte‐based (triphasic) processes is sparse. We believe that triphasic environments could provide opportunities for enhancing the performance of batteries, fuel cells, gas sensors and electrocatalysts by increasing local gas storage at electrodes, particularly if lessons from biological systems can be applied. The objective of this review is to provide a perspective to evaluate these opportunities.

### 1.1 Gas Storage/Transport in Biological Systems

**Figure 1:**
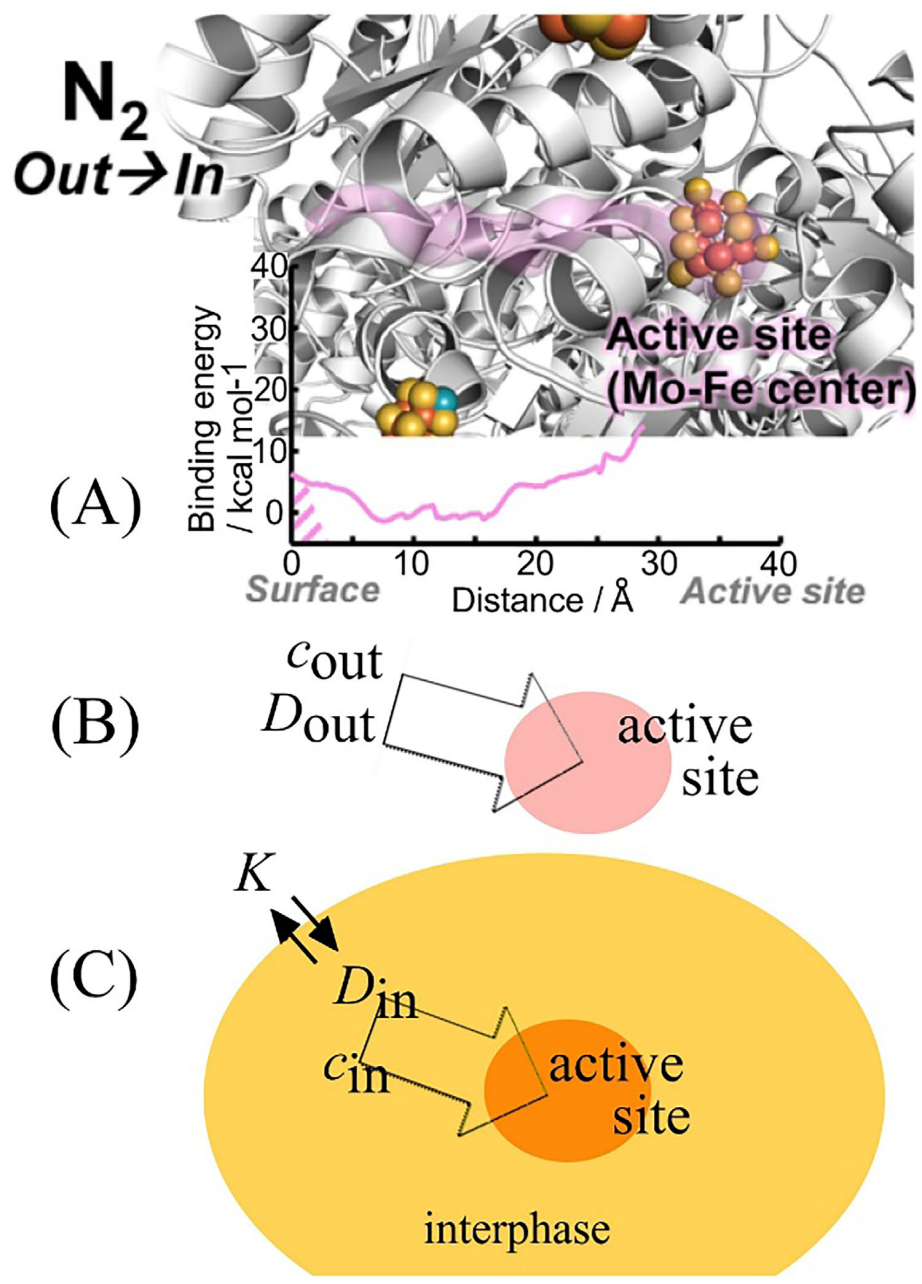
A) Illustration of a part of a nitrogenase structure with gas tunnel and binding parameters plotted as a function of distance (with permission^[^ ^24^ ^]^). B,C) Transport toward a reaction site without/with partitioning (with partitioning constant *K*) into an interphase surrounding the reactive site.

The inherently low solubility of many gases in aqueous environments presents a fundamental challenge to biological systems. Scenarios such as gases permeating through cell membranes^[^ ^18^, ^19^ ^]^ and gases transporting deep into the core of enzymes for biocatalysis^[^ ^20^, ^21^, ^22^ ^]^ have been studied extensively by experimentation and by computer simulation. In general, these processes involve the selective^[^ ^23^ ^]^/controlled transport of target gaseous species within precisely structured (evolved) pores, “gas tunnels” and cavities, for example, in enzymes (e.g., in nitrogenase or in myoglobin in Ref. [19]). Minor changes in the gas channel molecular structure completely change enzyme performance (**Figure** **1**A).

In a very oversimplified manner (using a Nernstian diffusion model in Ref. [25]), flux toward the reaction centre can be illustrated by binding into an interphase surrounding an active reaction site. Parameters *c*_out_ and *D*_out_ represent the flux limited by transport without interphase (Figure 1B). Then, an interphase (see Figure 1C) can be introduced with partitioning constant *K*, *c*_in_, and *D*_in_. Assuming first‐order conditions and partitioning (with free diffusion only in the unbound state), it is possible to express an approximate flux toward the active reaction centre. Partitioning constant *K* and apparent diffusion coefficients are defined in Equation (1).

(1) $$c_{in}=K\;c_{out}\;\mathrm{and}\;D_{in}=\frac{1}{1+K}\;D_{out}$$

(2) $$\mathrm{flux}\;\mathrm{to}\;\mathrm{the}\;\mathrm{reaction}\;\mathrm{site}\;\propto \;c_{in}\;D_{in}=\frac{K}{1+K}\;c_{out}D_{out}$$

This suggests that the apparent concentration and diffusivity in the vicinity of the reaction centre can be tuned with the help of the binding constant *K*. Enhancements are predicted for mass transport‐limited processes (for high *K* approaching the limit defined by *c*_out_ *D*_out_). For kinetically limited processes, reaction rates can be similarly tuned or even further enhanced (depending on reaction order) due to *K* affecting the apparent *c*_in_ and the corresponding local activity. The complexity of natural gas tunnel engineering in biological systems is high. Figure 1A shows an example of a variation in substrate (N_2_) binding as a function of distance from the reaction site. Biomimetic systems can be less complex, for example, based on microporous interphase materials in the vicinity of a catalyst or electrode. As will be described, some synthetic materials, such as metal–organic frameworks (MOFs) or intrinsically microporous polymers (PIMs) could provide “artificial gas tunnels” in interphases at electrode surfaces.

“Buried” active sites have been identified in many gas‐converting enzymes, including vital biocatalysts such as carbon monoxide dehydrogenase (CODH, for CO),^[^ ^26^ ^]^ formate dehydrogenase (FDH, for CO_2_),^[^ ^27^ ^]^ hydrogenase (for H_2_),^[^ ^28^, ^29^ ^]^ and nitrogenase (for N_2_).^[^ ^30^ ^]^ The specificity of these gas tunnels has been attributed to different characteristics, including selectivity based on polarity, molecular size and hydrophilicity. Tomita et al.^[^ ^22^ ^]^ described the process as a breathing motion, where the binding of CO molecules into a well‐studied protein (myoglobin) causes structural deformation of internal cavities. The fluctuating structure then “squeezes” the CO molecules to push them into a certain direction, apparently resulting in unidirectional transport of CO. Singh and Anand reported a similar observation and termed the behaviour pulsation mechanism.^[^ ^20^ ^]^ The binding of gas molecules as ligands in such cavities can be seen as a type of local storage.^[^ ^31^, ^32^ ^]^

Similarities between these biological systems and the microporous material‐based triphasic gas storage systems are evident. Both, fundamentally, rely on the creation of specialised micro‐environments, which modulate the local behaviour of gaseous species (e.g., the apparent diffusion coefficients), and enhance their local concentration. For this review, perhaps the most interesting question is linked to the engineering of these gas tunnels for improved performance. Bioengineering approaches aim to take the enzymes out of their natural environments and employ them in industrial applications, e.g., gas‐converting enzymes have the potential to offer sustainable and cost‐effective alternatives to noble‐metal catalysts in biofuel production, environmental restoration, carbon capture and nitrogen fixation.^[^ ^33^ ^]^ By selectively modifying the gas tunnels, researchers have been able to improve enzyme stability and efficiency in the presence of impurities – which is a vital issue for industrial applications.^[^ ^34^, ^35^ ^]^ In addition, efforts have been made to improve the efficiency of enzyme activities. Kim et al. demonstrated that a single point change in the tunnel composition can reduce the O_2_ sensitivity, a major bottleneck for CODHs in CO conversion, by a factor of 100.^[^ ^36^ ^]^ Moreover, the modification of the gas tunnel radius or chemical properties has been shown to alter the rate of diffusion of gaseous species, including the acceleration of transport of certain substrates.^[^ ^37^, ^38^ ^]^

Microporous materials for applications in the generation of a triphasic interphase (“gas tunnels”) with the ability to store gases in aqueous media can be chosen from carbons,^[^ ^39^ ^]^ porous metals,^[^ ^40^ ^]^ zeolites,^[^ ^41^ ^]^ microporous organic polymers,^[^ ^42^ ^]^ and metal–organic frameworks (for example, in metal‐gas batteries^[^ ^43^ ^]^). In particular, gas hydrates in porous materials are of wider interest.^[^ ^44^ ^]^ In recent studies by Erdosy and coworkers^[^ ^5^, ^45^ ^]^ the ability of intrinsically microporous materials such as silicate‐1 to store gases directly in aqueous environments (to give “microporous water”) has been highlighted. Here, two classes of microporous materials (both with considerable importance in electrochemistry and in the storage of hydrogen gas) are considered: metal–organic frameworks (MOFs; essentially crystalline systems with well‐defined micropore structure) and polymers of intrinsic microporosity (PIMs; amorphous systems with rigid “glassy” backbone and statistically defined ultramicropore or cage structure).

### 1.2 Metal–Organic Frameworks (MOFs)

**Figure 2:**
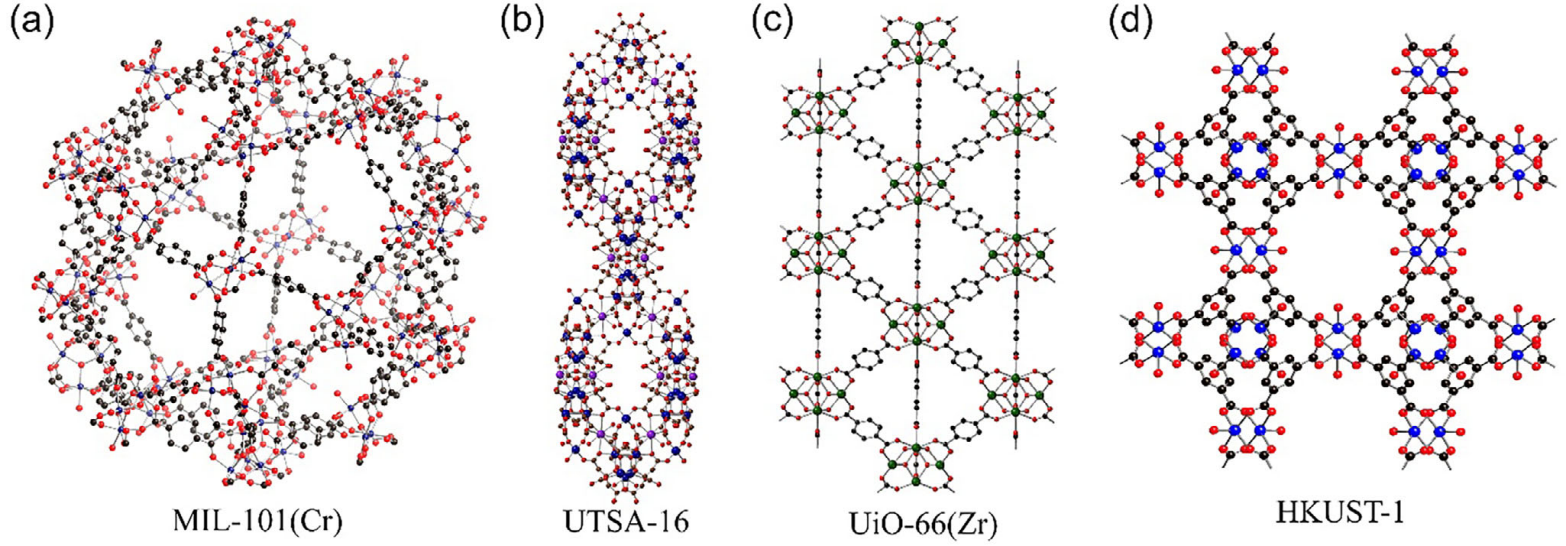
Framework structures for metal–organic frameworks a) MIL‐101(Cr)^[^ ^64^ ^]^ (Cr_3_O(OH)(H_2_O)_2_(bdc)_3_, where bdc = 1,4‐benzenedicarboxylate), b) UTSA‐16^[^ ^66^ ^]^ (K_2_Co_3_(citrate)_2_), c) UiO‐66(Zr)^[^ ^68^ ^]^ (Zr_6_O_4_(OH)(bdc)_6_), d) HKUST‐1^[^ ^69^ ^]^ (Cu_3_(btc)_2_(H_2_O)_3_, where btc = 1,3,5‐benzenetricarboxylate).

The general structure for MOFs consists of metal ions or clusters with organic ligands linking them, forming large, interconnected, and usually crystalline networks that may be 2 or 3D in nature (see **Figure** **2**).^[^ ^46^, ^47^ ^]^ MOFs have been suggested for many applications including energy technologies,^[^ ^48^ ^]^ water harvesting^[^ ^49^ ^]^ and for carbon dioxide capture/conversion.^[^ ^50^ ^]^ New types of MOFs, such as MOF‐303 promise better compatibility and stability with water.^[^ ^51^ ^]^ The applications of MOFs are strongly linked to adsorption into extended microporous structures with sometimes massive and tuneable internal surface areas,^[^ ^52^ ^]^ which also make them extremely well suited to triphasic gas or small molecule storage.^[^ ^53^, ^54^ ^]^ Many MOFs are known with a range of pore size, metal oxidation state, and functionalisation of organic linkers,^[^ ^55^ ^]^ but despite the extensive research on their applications, there is little known on their ability to create a triphasic (aqueous) environment for electrocatalysis.

Metal–organic frameworks (MOFs) may be considered as crystalline inorganic counterparts of amorphous PIMs, due to the shared properties of high porosity, high internal surface areas, and tuneability and range of compounds. The choice of metal and organic components, and conditions of synthesis, tune the properties of the resultant MOF: for example, the length, shape, and rigidity of organic linkers will affect the crystal structure and therefore the porosity of the material. Many MOFs can store gases under dry conditions, and they often have high surface areas (up to 10 000 m^2^ g^−1^), surpassing classical conventional gas storage materials such as zeolites and activated carbon. MOF structure or synthesis may be changed to affect the chemical and thermal stability, pore size, surface area, structure, and catalytic activity. Some MOFs are now available on a large scale (such as commercially available Basolites in Ref. [56]), and novel techniques to repurpose plastic waste into organic ligands for MOFs indicate the many potential avenues for industry‐scale MOF synthesis.^[^ ^57^ ^]^

Due to their high surface area and porosity, allowing for extensive gas storage, many MOFs have been reported for hydrogen storage, allowing green, efficient energy storage for a hydrogen economy.^[^ ^58^, ^59^ ^]^ MIL‐101(Cr) (Figure 2a) offers high hydrogen capacity (vide infra), and stability toward heat, moisture, solvents, and air over months, making it robust and suitable for hydrogen storage over a range of conditions.^[^ ^60^ ^]^ Novel synthesis methods aimed at making large‐scale use economically viable have been reported, such as a MIL‐101(Cr) one‐pot synthesis that uses recycled PET plastic to form organic linkers.^[^ ^57^ ^]^ This method could allow sustainable large‐scale production, reducing the upfront cost of hydrogen storage. MOFs can store many types of gases, a feature which allows application in green technologies such as CO_2_ capture. CO_2_ is stored in MOFs by adsorption, allowing degassing by heating to free up the adsorbent, and allowing more space‐efficient storage compared to compressed gas cylinders.^[^ ^61^ ^]^ MOF materials such as UTSA‐16 (Figure 2b ^[^ ^62^, ^63^ ^]^) have been 3D‐printed into membrane structures. Water‐stable UiO‐66(Zr) materials (Figure 2c) have attracted attention for applications in water‐based technology.^[^ ^64^ ^]^ The HKUST‐1 cage‐type material (Figure 2d) has been shown to provide a high performance in gas adsorption applications.^[^ ^65^ ^]^

### 1.3 Polymers of Intrinsic Microporosity (PIMs)

**Figure 3:**
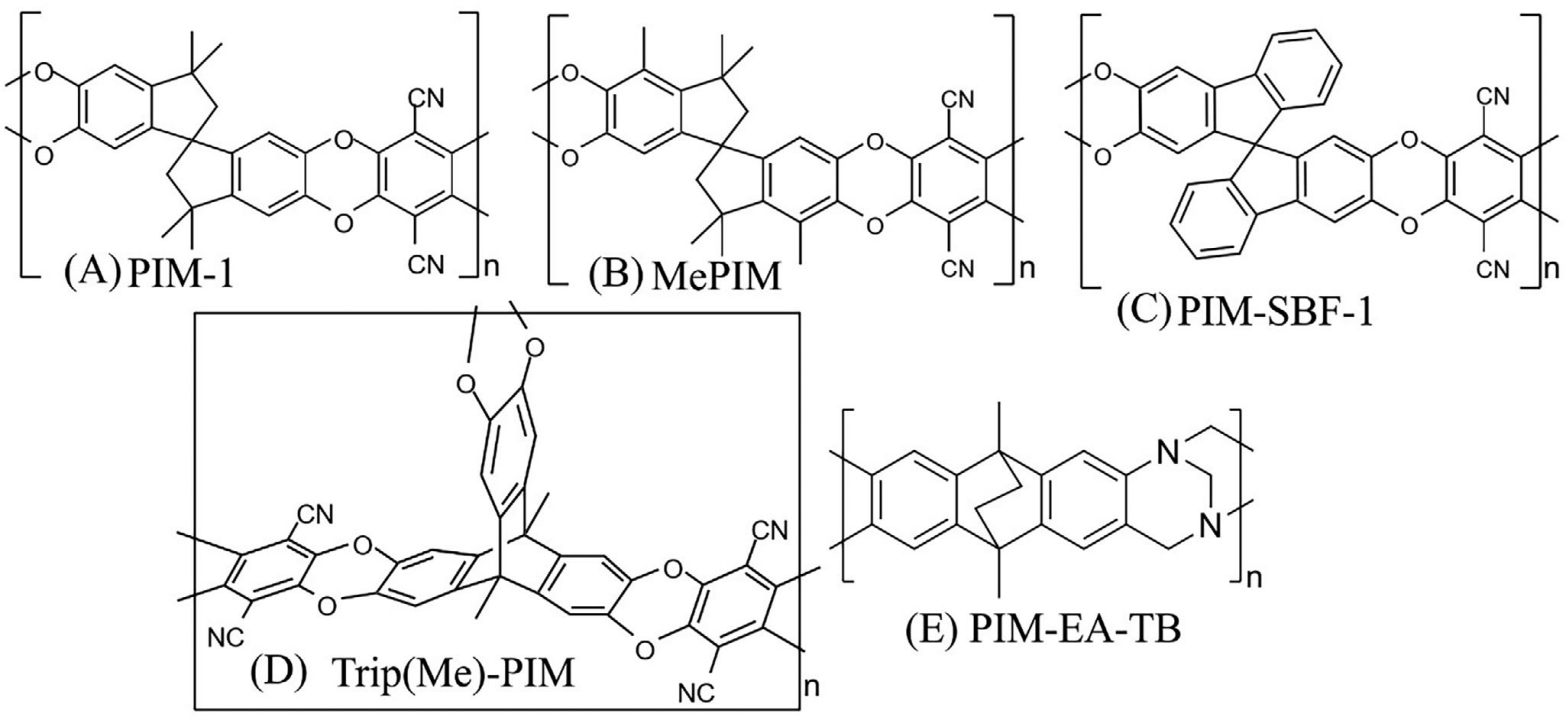
Molecular structures for A) PIM‐1,^[^ ^74^ ^]^ B) MePIM,^[^ ^75^ ^]^ C) PIM‐SBF‐1,^[^ ^76^ ^]^ D) Trip(Me)‐PIM,^[^ ^77^ ^]^ and E) PIM‐EA‐TB.^[^ ^75^, ^78^ ^]^

PIMs, owing mainly to their combination of processability and intrinsic microporosity, have a wide range of potential applications, particularly as membrane materials.^[^ ^66^ ^]^ The properties of PIMs are due to the rigidity and contortion of its macromolecules together with its lack of conformational freedom due to a fused‐ring structure. For example, PIM‐1 (**Figure** **3**) is constructed only of fused rings and spirocyclic units resulting in contorted macromolecular chains that pack very inefficiently in the solid state, which creates a network of micropores,^[^ ^67^ ^]^ suitable for gas binding (biphasic or triphasic).^[^ ^68^ ^]^ PIM structures may vary, but generally consist of contorted structural units (e.g., fused ring systems, spirocyclic units) and rigid linking motifs (often dibenzodioxin, bridged bicyclic amines, or imide bonds around which rotation is restricted, Figure 3).^[^ ^69^ ^]^ There are significant differences in internal hydrophobicity depending on heteroatoms and chemical motifs.

An advantage of PIMs is the eponymous intrinsic microporosity, meaning the ability of PIMs to store gases or create an environment that does not excessively degrade over time. For example, PIM‐1 maintains its mechanical properties indefinitely, which indicates that PIMs are suited to long‐term applications. The long‐term stability of PIM‐1 was studied as a dry film^[^ ^70^ ^] –^ it is possible that constant immersion in an electrolyte (like in a fuel cell) will alter the chemical and mechanical stability of the PIM coating, and further testing of PIMs mechanical stability depending on reaction conditions should be done to quantify this.

Most PIMs are soluble in organic solvents, such as chloroform or THF—their rigid and contorted macromolecular structure limits inter‐chain interactions and thereby increases solubility. This is likely a twofold effect: poor solid‐state packing decreases inter‐chain contact, which subsequently weakens intermolecular interactions between the chains, and the microporosity allows good penetration of a solvent into the structure. Their solubility makes PIMs easily processable: they can be cast into membranes or films without difficulty,^[^ ^71^, ^72^ ^]^ making them well‐suited to large‐scale applications. Once a solution is prepared, it can be applied to an electrocatalyst simply by drop‐casting or spin‐coating, which is useful for scaling up. Despite the many beneficial physical and chemical properties of PIMs, the economics of their use still present an issue. The price of commercially available PIMs currently ranges from £200/g (PIM‐1) to £750/g (PIM‐EA‐TB), although larger‐scale industrial synthesis would reduce these costs considerably.^[^ ^73^ ^]^

## 2 Microporous Materials for Biphasic Processes at the Gas|Solid Interface

In order to discuss gas binding phenomena under triphasic conditions, it is helpful to first consider biphasic conditions, where gases are bound directly to solid microporous materials. The US Department of Energy (DoE) has provided an ultimate target H_2_ uptake value for automotive storage (a major part of a hydrogen fuel economy) of 6.5 wt.%, and also provides a value indicative of the current state of hydrogen storage: for adsorption‐based methods, this is 3.8 wt.% (from MOF‐5 at 80 K and 100 bar).^[^ ^85^ ^]^ These values are useful benchmarks to assess the efficacy of porous materials for dry hydrogen storage.

### 2.1 Dry Gas Storage in Metal–Organic Frameworks and Composite Materials

Many MOFs have been studied for gas storage. One example is MIL‐101(Cr), a chromium‐based MOF first synthesised by Ferey et al. known for its stability to heat and humidity, as well as its exceptional surface area (4230 m^2^ g^−1^) and “giant pores” (pore volume of MIL‐101 is typically 2.15 cm^3^ g^−1^).^[^ ^79^, ^80^ ^]^ Due to this, MIL‐101 has seen extensive research for gas storage, and has an impressive hydrogen capacity of 6.1 wt.% (80 bar, 77 K).^[^ ^81^ ^]^ This gas binding property is likely due to the presence of microporous super‐tetrahedra (tetrahedra constructed from smaller tetrahedra) formed by the MIL‐101 structure, intuitively, these super‐tetrahedral sites may also be advantageous for triphasic gas storage by providing a base for nanobubble formation.

Composites of MOFs with other porous materials, imparting desirable properties such as conductivity, surface area, porosity, and handling, have been reported. A number of studies have considered PIM‐1 as a binding agent with MOFs or other highly porous materials.^[^ ^82^, ^83^ ^]^ These composites benefit from the interconnection of microporous networks in the constituent materials, providing increased surface area and porosity for gas storage capabilities: some composites will exhibit a decrease in overall specific surface area, as is the case for the UiO‐66(Zr)/PIM‐1/CF (carbon foam) composite reported by Molefe et al.^[^ ^83^ ^]^ Good processability of the UiO‐66(Zr)/PIM‐1 mixture allowed preparation by impregnation, simply by soaking carbon foam in a suspension of the composite components. Although this is an effective and facile method for immobilisation of MOFs onto a substrate, there was poor penetration of UiO‐66(Zr)/PIM‐1 into the carbon foam, leading to a decrease in surface area (pure UiO‐66(Zr) = 1367 m^2^ g^−1^, UiO‐66(Zr)/PIM‐1/CF = 768 m^2^ g^−1^) and hydrogen uptake (pure UiO‐66(Zr) = 1.38 wt.%, UiO‐66(Zr)/PIM‐1/CF = 1.05 wt.%, both measurements at 1 bar, 77 K, see **Tables** **1** and **2** for PIM‐1 values). Higher MOF loadings may increase the penetration into the carbon foam substrate, thereby improving hydrogen capacity. The low density of carbon foam and the ease of preparation of this composite present a powerful hydrogen storage material, if the hydrogen capacity itself can be suitably increased (US DoE hydrogen storage target = 4.5 wt.%^[^ ^84^ ^]^).

**Table 1:** Summary of properties for MOF materials for dry hydrogen storage and the corresponding BET surface areas.

Metal–Organic Framework	BET surface area/m^2^ g^−1^	H_2_ uptake at 77 K/wt.%		Refs.
		1 bar	Max.	
MIL‐101	4230	1.8	6.1 (60 bar)	[60, 81, 82]
UiO‐ 66(Zr)/PIM‐ 1/CF	768	1.05	‐	[83]
UiO‐ 66(Zr)/PIM‐ 1/ZTC	1767	1.65	‐	[85]
PIM‐ 1/80 wt.%ZTC	2433	1.87	‐	[85]

**Table 2:** Summary of properties for PIMs for dry hydrogen storage and the corresponding BET surface areas.

Porous material	BET surface area/m^2^ g^−1^	H_2_ uptake at 77 K/wt.%			Refs.
		1 bar	10 bar	max.	
PIM‐1	760	1.04	1.44	‐	[87]
PIM‐1‐110‐15 h‐350‐4 h	436	1.15	1.6	3.67 (90 bar)	[88]
MePIM	921.6	1.18	‐	1.90 (55 bar)	[75]
Trip(Me)‐PIM	1760	1.80	3.2	3.4 (18 bar)	[77]
PIM‐1/PAF1(37.5)	1639	1.15	‐	4.79 (100 bar)	[92, 93]

Molefe et al. described UiO‐66(Zr)/PIM‐1/ZTC composites that incorporated 40 wt.% UiO‐66(Zr) for porosity; 20 wt.% PIM‐1 as a binder; and 40 wt.% zeolite‐templated carbon (ZTC) for thermal conductivity. Interestingly, the PIM‐1/80 wt.%ZTC composite without MOF had a higher hydrogen capacity than the UiO‐66(Zr)/PIM‐1/ZTC composite (1.87 and 1.65 wt.%, respectively). This suggests a pore blocking effect associated with the inclusion of MOF nanocrystals and PIM‐1, disrupting the interconnected porous network, affecting surface area; ZTC retains more of its pure properties when in composition, which helps to mitigate pore blockage.^[^ ^85^ ^]^

### 2.2 Dry Gas Storage in Polymers of Intrinsic Microporosity and Their Composites

PIMs exhibit moderate gas adsorption at low pressures: combined with their porosity, high surface area, and processability, this makes them ideal for gas storage. The hydrogen capacity of PIM‐1 has been reported in the literature multiple times: at conditions of 1 bar and 77 K the hydrogen capacity has been reported from 0.81 to 1.04 wt.%, rising to 2.60 wt.% under high‐pressure conditions (100 bar, 77 K).^[^ ^86^ ^]^ Evidently, these values fall short of the US DoE targets. A method to anneal PIM‐1 was reported, which involved heating a sample of PIM‐1 at 110 °C for 15 h, then 350 °C for a further 4 h. The resultant polymer, designated PIM‐1‐110‐15 h‐350‐4 h, gave hydrogen uptake values of 1.1 wt.% (at 1 bar, 77 K) and 1.6 wt.% (10 bar, 77 K). A high‐pressure measurement at 90 bar and 77 K showed hydrogen uptake at 3.6 wt.%.^[^ ^87^ ^]^ These promising values indicate that annealing polymer films could provide a simple method to increase hydrogen capacity: it would be informative to expose a range of PIMs to different annealing methods to gain a wider understanding of this effect. It is thought that the heat treatment increases the microporosity by removing other adsorbents that were filling micropores, freeing/modifying space for gas adsorption, however it is more likely that the annealing produces a greater number of ultramicropores, which may increase H_2_ uptake. Over time, it was found that the amount of hydrogen storage in a film of PIM‐1 decreased due to a gradual reduction in free volume (i.e, physical ageing): hydrogen uptake was initially measured at 2.6 wt.%, which decreased to 1.9 wt.% after 400 days had elapsed (measurements taken at 80 bar, 77 K).^[^ ^88^ ^]^ Although this loss of efficacy is not ideal, there are methods of functionalisation to modify PIM structures to increase hydrogen capacity,^[^ ^89^ ^]^ and more research into effective PIM structures could solve this problem.

Adding a methyl group to the spiro‐bisindane moiety to give the structure MePIM (Figure 3B) was found to slightly increase the hydrogen capacity to 1.18 wt.% (1 bar, 77 K), and a maximum value was attained at high pressure of 1.90 wt.% (22 bar, 77 K).^[^ ^75^ ^]^ The addition of the methyl group makes solid‐state packing more inefficient, which increases the micropore volume and surface area, subsequently increasing the hydrogen capacity. Additionally, although MePIM retains its solubility, which is useful for processability, it was reported that films were noticeably more brittle during handling, which limits applications.^[^ ^75^ ^]^

Other PIMs have been reported with even greater hydrogen capacity, such as the triptycene‐derived network polymer Trip(Me)‐PIM (Figure 3D), which gives excellent hydrogen uptake values of 1.80 and 3.2 wt.% (1 bar and 10 bar, respectively, 77 K), with a maximum value of 3.4 wt.% (18 bar, 77 K). It is thought that the triptycene moieties increase microporosity by forcing inefficient space packing. Longer alkyl chains in place of the methyl groups are more flexible and will be free to move to occupy empty pores, subsequently decreasing microporosity: this allows tuning PIM micropore size.^[^ ^77^ ^]^ Due to its increased microporosity, the hydrogen capacity of Trip(Me)‐PIM is far higher than most other known PIMs.^[^ ^77^ ^]^ Despite this, it is less suited for many applications on account of its insolubility. This makes processing the polymer highly inconvenient and, for triphasic applications, raises the challenge of how to deposit Trip(Me)‐PIM onto an electrode surface. Solubility is a critical property for microporous materials in electrochemical applications, where triphasic storage is most useful.

PIMs may have their gas storage enhanced by mixture with other porous materials to form composites, often with more desirable properties than each on their own, as shown by Mays and co‐workers.^[^ ^90^ ^]^ For example, composites of PIM‐1 and a porous aromatic framework (PAF‐1), an insoluble organic network polymer of high surface area, were reported and exhibited exceptional hydrogen uptake values: the best performing composite had a hydrogen capacity of 1.15 wt.% (1 bar, 77 K) and a high‐pressure hydrogen capacity of 4.79 wt.% (100 bar, 77 K).^[^ ^91^ ^]^ PAFs are largely rendered in powder form, which limits their processability compared to PIMs; however, by compositing with the solution processable PIM‐1, the higher surface area of PAF‐1 and the processability of PIM‐1 are combined. Multiple composites were made with different loadings of PAF‐1^[^ ^92^ ^]^ and it was evident that increasing the loading of PAF‐1 correlates with a higher internal surface area and hydrogen capacity, but also with a decrease in the amount of tensile stress the resulting composite films could withstand. Crucially, a “tipping point” was determined at 20 wt.% PAF‐1, where the films become brittle and difficult to handle practically, often cracking during preparation. Therefore, a balance between hydrogen uptake and robustness was found: the highest hydrogen capacity was associated with a composite containing 37.5 wt.% PAF‐1, and although still resulting in a “*robust and self‐standing film*,” no mechanical testing was performed on it. Molecular modelling, instead of time‐consuming systematic approaches, has seen limited use in the development of new PIM structures,^[^ ^93^ ^]^ and its use could be expanded to composites.

It is important to note that in addition to the storage of hydrogen gas (some PIMs mentioned in this review for hydrogen storage are summarised in Table 2), other gases are adsorbed into PIMs, interacting differently and diffusing through at different speeds.^[^ ^94^ ^]^

The extent of dry storage (or of triphasic storage) is not the same for different gases, for example: for triphasic storage, *c*_app,hydrogen_ increases from *≈*0.08 to 80 mm, an increase of ≈1000 times, whilst *c*_app,oxygen_ increases from *≈*0.3 to 50 mm, an increase of ≈160 times.^[^ ^11^, ^95^ ^]^ Despite the same PIM‐1 nanoparticle film thickness, the different molecule sizes and properties affect transport through the micropores and binding to the microporous host. Characterisation methods such as adsorption isotherms, thermogravimetric analysis, and electrochemical probing could be employed to better understand the interactions of gases with microporous networks to optimise storage for different gases.

In the case of CO_2_ adsorption into microporous carbon, beneficial effects of humidity were reported,^[^ ^96^ ^]^ but in other types of microporous systems the opposite trend was observed.^[^ ^97^ ^]^ Generally, one would expect humidity to compete with gases for surface binding sites under triphasic conditions, but the high surface tension of water could also enhance gas binding to lower surface energy. Gas molecules can replace water at the solid|liquid interface and in this way lower surface energy. Interfacial bubbles can form to store gas in the presence of liquid. Therefore, it is interesting to explore triphasic gas binding in the presence of liquid water.

## 3 Microporous Materials for Triphasic Processes and Interfaces I.: Storage

Materials with bound oxygen gas are known to enhance electrochemical oxygen reduction responses. The Clark oxygen sensor,^[^ ^98^, ^99^ ^]^ which can be employed for oxygen as well as for hydrogen,^[^ ^100^ ^]^ is based on a Teflon membrane in close vicinity (within the diffusion layer) to a platinum disk electrode. Gases permeate and are transported across the diffusion layer to allow quantitative detection. Fluorinated materials and Teflon (PTFE) are known to bind/permeate oxygen and other gases, with applications such as emergency blood replacement,^[^ ^101^ ^]^ although one could argue that molecular solubility is not necessarily linked to triphasic storage.

PIMs may store gas under dry conditions as well as in a triphasic environment. Intuitively, materials with a high hydrogen/gas uptake should be able to store more gas at an electrode surface, thus further increasing apparent concentration, so will be suitable for triphasic storage. This relationship between dry and triphasic storage capabilities is yet to be experimentally established. However, by comparing the apparent concentration of hydrogen and hydrogen adsorption isotherms, a relationship can be approximated. The experimentally observed apparent concentration of hydrogen in a triphasic environment containing PIM‐1 nanoparticles, *c*_app,hydrogen_ = 80 mm, corresponds to a pure gas concentration at a pressure of 2 bar of pure hydrogen.^[^ ^95^ ^]^ By comparing to a hydrogen adsorption isotherm, a pressure of 2 bar indicates an expected hydrogen uptake value for triphasic hydrogen at 1.2 wt.%. This value, recorded at ambient temperature, is higher than the corresponding dry hydrogen uptake values of PIM‐1 measured at 77 K, showing the potential for increased capacity for gas storage in triphasic systems.

PIMs in aqueous environments are known to accumulate not only gases but also hydrophobic molecules in the pores. In a study based on the electrocatalytic oxidation of alcohols, it was shown that partitioning of more hydrophobic alcohols into a PIM‐EA‐TB host film (in water) increased the rate of oxidation.^[^ ^102^ ^]^ Both uptake of hydrophobic guest species and gaseous guests are linked to hydrophobicity and surface tension effects in pore water. The ability of PIMs to capture and store both hydrogen and oxygen in triphasic environments could provide an opportunity for rechargeable hydrogen/oxygen batteries, as both these gases evolve and must be stored close to the electrode during their use. By depositing PIMs onto electrode surfaces, the increase in apparent concentration of hydrogen enhances certain electrochemical processes. In particular, electrochemical probing reveals that in the presence of PIM‐1, additional peaks for hydrogen oxidation become visible on successive cyclic voltammograms. The oxidation of hydrogen and the reduction of oxygen stored in the triphasic environment in the polymer (vide infra) can be enhanced. The oxygen reduction peak is shifted to a more positive potential, indicative of an enhanced reaction pathway that increases the process.^[^ ^68^ ^]^

PIM films can enhance gas‐evolving/consuming reactions and could be used more widely. The nitrogen reduction reaction and the carbon dioxide reduction reaction, two potentially green electrochemical processes to produce ammonia and carbon feedstocks, respectively, have both been shown to be enhanced by the creation of triphasic environments.^[^ ^103^, ^104^ ^]^ Similarly, the electrochemical reduction of CO_2_ using a nanoporous copper catalyst was reported/reviewed to have a respectable selectivity for desired high‐value multi‐carbon products.^[^ ^105^ ^]^ The presence of micropores likely induces selectivity and alters efficiency by affecting the diffusivity and apparent concentration of gases within the triphasic environment. PIMs are highly suited to triphasic storage in these applications, especially as they are shown to have far higher surface areas than the catalysts (typically, PIM‐1: 760 m^2^ g^−1^, Pd/activated C: 96 m^2^ g^−1^, nanoporous Cu: 7.02 m^2^ g^−1^), and their processability makes them attractive for large‐scale industry applications.^[^ ^106^ ^]^

Many PIMs have been developed specifically for hydrogen storage applications.^[^ ^107^ ^]^ PIMs and associated composites are suited for use as membranes in gas separation, which is a sustainable, pressure‐driven technology that separates gases by their differing permeability (flux) in a material.^[^ ^108^ ^]^ PIMs, due to their rigid shape and interconnected microporous networks, provide excellent gas permeability and provide gas selectivity based on micropore size. Selectivity between certain gas pairs may be tuned by the alteration of the polymer and the creation of composite membranes, opening up a wide range of PIMs for this application. PIM‐1, as the classic example of a PIM, is effective for a multitude of separation applications, such as CO_2_ capture,^[^ ^109^ ^]^ and fluorinated gas capture,^[^ ^110^ ^]^ while composites have been shown to increase the selectivity and maintain high gas permeability of O_2_/N_2_ separation.^[^ ^111^ ^]^

A major issue associated with the creation of triphasic environments using microporous materials is the slower diffusion of gases to and from the electrode surface, in part due to the poor solubility of gases in aqueous media, and in part due to the adsorption of gas molecules into rigid micropores with low diffusivity. Despite this, there are potential benefits of triphasic environments in electrocatalysis: microporous materials can conceivably be used in a range of electrochemical processes to increase their effectiveness, including processes in fuel cells, N_2_ reduction, CO_2_ reduction, water electrolysis, battery processes, pollution treatment, electrolysis, and processes in electroanalytical sensing applications.^[^ ^112^ ^]^

### 3.1 Microporous Materials Affecting Apparent Gas Concentration in Water

**Figure 4:**
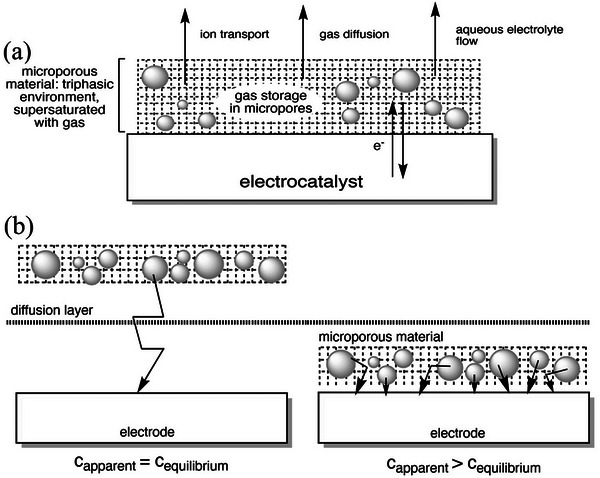
a) Illustration of triphasic gas storage and transport in/out a microporous material at an electrode surface. b) Contrasting (left) the case of gas storage outside of the diffusion layer and (right) gas storage inside the diffusion layer.

The mechanism by which microporous materials increase the apparent concentration of gases at electrode surfaces is inherently linked to the creation of the triphasic environment. Therefore, an understanding of these environments is crucial to evaluating the effectiveness of microporous materials in fuel cells or batteries. First, gases adsorb into the micropores of such materials by the process of physisorption; the primary intermolecular forces present are van der Waals interactions.^[^ ^113^ ^]^ Aqueous electrolytes partially flow into the micropores, creating a three‐phase system with trapped bubbles (gaseous reactant/product, aqueous electrolyte, solid microporous material). This triphasic domain stabilises gases by the formation of nanobubbles, but it also limits gas diffusion due to partial adsorption of gases into the micropores. A “supersaturated” region of gas forms within the microporous material where gas may be stored as adsorbent or in nanobubbles, leading locally to the increased apparent concentration of gases (**Figure** **4**). These nanobubbles do not block active sites (as in the absence of microporous materials) as might be expected, because they interact with the microporous material, rather than directly adhering to the electrode surface. However, the microporous material must be within the diffusion layer (Figure 4b) to have any effect. An increased apparent concentration of gases promotes the corresponding reaction, leading to an apparently greater electrocatalytic activity.

The triphasic storage ability of microporous materials in electrochemical cells may be quantified by the apparent concentration, *c*_app_, and the apparent diffusion coefficient, *D*_app_, which are distinct from the corresponding values for homogeneous solutions. For the case of a chronoamperometry experiment in homogeneous phase and with a time‐dependent diffusion layer, the Cottrell equation^[^ ^114^ ^]^ applies (Equation 3). In the presence of microporous material, a similar equation (Equation 4) can be expressed, but the meaning of *c*_app_ and *D*_app_ is now mechanistic, case‐dependent. To state this in a different way, for example, in cyclic voltammetry experiments, the current peak could be decreased although the true concentration of gaseous species could be much higher. Both concentration and diffusion coefficient require attention. Unravelling each of the contributions requires multiple experiments.

(3) $$I\left(t\right)=\frac{\mathrm{nFAc}\sqrt{D}}{\sqrt{\pi t}}\;$$

(4) $$I\left(t\right)=\frac{\mathrm{nFA}c_{app}\sqrt{D_{app}}}{\sqrt{\pi t}}\;$$

Here, *n* is the number of electrons transferred per molecule diffusing to the electrode, *F* is the Faraday constant, *A* is the electrode area, *c* is the bulk concentration, *D* is the diffusion coefficient in solution, and *t* is the time. When a microporous material is located outside of a diffusion layer (Figure 4b), the stored gas dissolves into the electrolyte. The availability of gases to the electrode is independent of the distance between the microporous material and the electrode and is simply based on the homogeneous/equilibrium concentration, which in this circumstance is equal to the bulk concentration. If a microporous material is within the diffusion layer of an electrode (Figure 4b), gas transport is enhanced, so there is a higher gas flux to the electrode that is not otherwise present, and the apparent concentration (i.e., how much gas is available to the electrode) increases. Therefore, apparent concentration is used to quantify the kinetic availability of gases based on diffusion speed, in addition to the thermodynamic aspect of adsorption within micropores; a higher apparent concentration correlates to an increased rate of reaction, because more reactant is available to the catalytic site. Note, there are many mechanistic sub‐cases, and the Cottrell equation may not always be appropriate.

### 3.2 Triphasic Gas Storage in Metal–Organic Frameworks

Erdosy et al. have reported a method to increase the solubility of gases in aqueous media, by creating “microporous water”: nanocrystals of a porous zeolite (silicalite‐1 or a MOF) dispersed within water, with hydrophilic external surface chemistry to promote dispersion, and hydrophobic internal surface chemistry to ensure that water is thermodynamically dissuaded from entering the microporous network, leaving the space free for gases to compete for adsorption sites.^[^ ^4^, ^5^ ^]^ The concept of liquids with permanent, intrinsic microporosity is not a new one, indeed, microporous water may be thought of as a Type 3 porous liquid: a solid microporous framework dispersed in a solvent.^[^ ^115^ ^]^ The key difference is that classic Type 3 porous liquids (like all previous porous liquids) require a sterically hindered solvent that is too large to enter network pores, to promote gas adsorption. The thermodynamic approach of Erdosy and co‐workers prevents small molecule solvents like water from entering pores, opening up applications in biomedical technologies for gas transport, and a wide array of electrochemical technologies: many important green technologies (nitrogen reduction, oxygen reduction, hydrogen evolution, carbon dioxide reduction) benefit from increased gas solubility in aqueous media. The use of a 6.7 vol.% solution of silicalite‐1 nanocrystals in water as an aqueous electrolyte was described for the oxygen reduction reaction, resulting in an ≈4× increase in current density (vide infra). There is direct correlation between the concentration of silicalite‐1 nanocrystals in solution and current density.^[^ ^4^ ^]^ The increased current density is likely due to an increased apparent concentration within the diffusion layer, leading to higher gas flux to the electrode, suggesting a triphasic storage interaction. In the same way that gaseous nanobubbles will associate with a microporous material to avoid blocking interactions with an electrode and maximise three‐phase interface zones, gases adsorb into the free pores of nanocrystals of silicate‐1, associating with the microporous network there. A triphasic interaction arises from the solid nanocrystal, the stored gas, and the water at the mouth of the pores. It is important to note that free gas molecules are also dissolved in the water. Finding the cause of increased activity is complex in a dynamic system such as this, but it is likely a result of the combination of increased gas solubility and diffusion from within the diffusion layer, leading to higher gas flux to the electrode.

**Figure 5:**
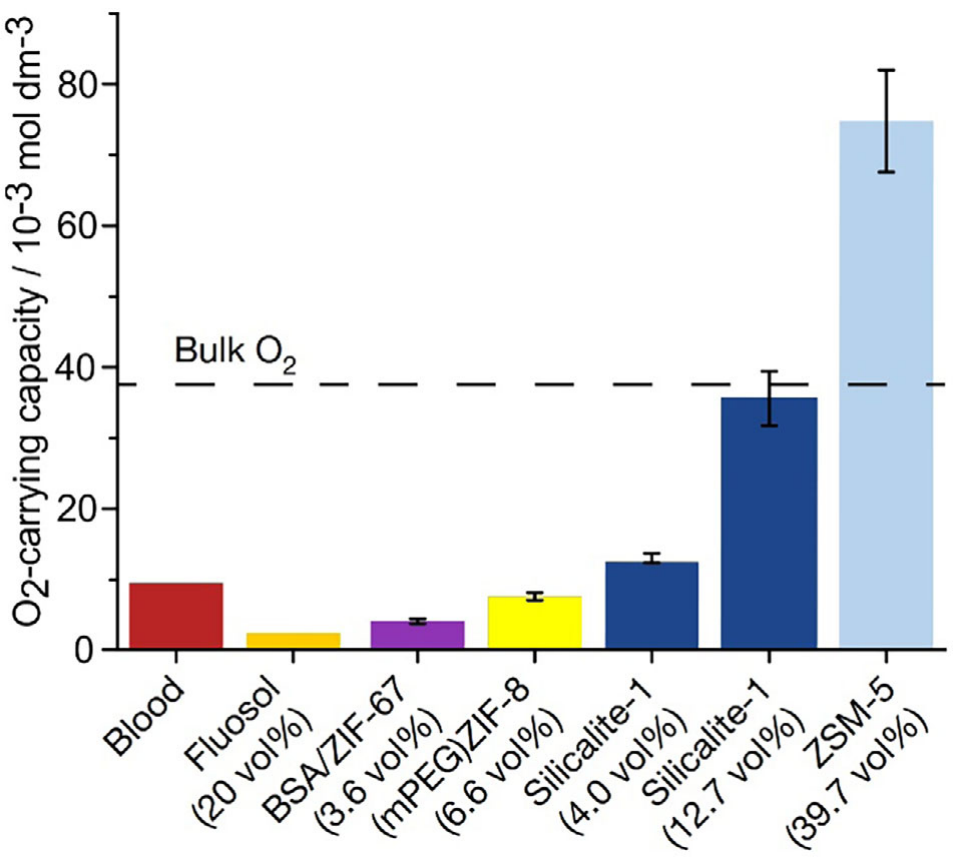
Comparison of the O_2_‐carrying capacities of aqueous solutions of hydrophobic zeolite and MOF nanocrystals to the O_2_‐carrying capacities of blood (i.e., 150 g Hb dm^−3^) and a representative perfluorocarbon emulsion (Fluosol). All capacities are for aqueous solutions equilibrated at 1 bar O_2_ near ambient temperature (adapted with permission^[^ ^5^ ^]^).

Although silicalite‐1 is not an MOF, microporous water with zeolitic imidazolate framework (ZIF, a subset of MOFs) nanocrystals have demonstrated the same ability to increase gas solubility.^[^ ^5^ ^]^ In fact, due to possessing generally higher surface areas than zeolites, MOFs are arguably better suited to microporous liquids, and the mechanism is the same irrespective of whether the nanocrystal is a zeolite or MOF. Molecular dynamics simulation methods have been used by Erdosy et al.^[^ ^5^ ^]^ to show gas replacing water in hydrophobic cavities as a plausible mechanism for storage. A summary of triphasic gas storage effects for oxygen in different types of microporous ZIFs, in silicate‐1, and in the zeolite ZSM‐5 is shown in **Figure** **5**. In comparison to oxygen in pure water (typically 1.2 mm), oxygen in blood can reach 25 mm concentration (Figure 5). Fluosol as an oxygen carrier, as well as ZIF‐67 and ZIF‐8‐based suspensions, remain below this gas storage value. Data for silicate‐1 and for ZSM‐5 suggest substantially higher storage of oxygen gas in micropores. The dashed line corresponds to pure oxygen gas in the gas phase (1 bar, ambient temperature).

Further research should focus on quantifying the performances of MOF nanocrystals: density measurements to confirm pores free of solvent, NMR studies of guest species in pores, and rotating disk electrode experiments for current density in the manner of Erdosy et al. would be particularly useful.^[^ ^4^, ^5^ ^]^ Approaches to control the size of MOF nanoparticles are well established^[^ ^116^ ^]^ and nanoparticle size can affect guest uptake and release (both the rate and the efficiency).^[^ ^117^ ^]^

The development of humidity‐resistant MOF/PIM composite membranes for gas separation may be an example of triphasic gas storage, enhancing membrane separation processes. Zhou et al. described functionalisation of a UiO‐66 MOF for CO_2_/N_2_ separation with hydrophobic trifluoromethyl groups (giving UiO‐66‐CF_3_), creating hydrophobic surface chemistry, whilst allowing water molecules to enter the micropores, essentially the inverse of the microporous liquids of Erdosy and coworkers. The hydrophobic groups increase the water stability of MOFs, solving a long‐known issue.^[^ ^118^ ^]^ With a water‐stable MOF, the effects of humidity become visible, and a triphasic interaction is likely. UiO‐66‐CF_3_ was combined with PIM‐1 to act as a matrix, to give a composite membrane (mixture of UiO‐66CF_3_/PIM‐1, polydimethylsiloxane, and polysulfone), imparting uniquely useful qualities to the membrane. CO_2_ permeability in dry conditions is enhanced by a number of factors: polar interactions between ‐CF_3_ and CO_2_, polar interactions between ‐CN (on PIM‐1) and CO_2_, and the unique topology of the mixed membrane. The micropores form an interconnected network between PIM and MOF, the channels of which are selective by polar interactions and by size exclusion (nitrogen molecules are larger than carbon dioxide molecules). When the water vapour content was increased to 10% to simulate a humid environment, the CO_2_/N_2_ selectivity of a membrane with an 8% loading of UiO‐66‐CF_3_ increased from 33.8 to 43.7. The reason for this increase is linked to the reduction in the nitrogen permeability, due to competition with water molecules filling the micropores, whilst carbon dioxide permeability is largely unchanged due to the stabilising polar interactions within the network. However, it is also possible that within the pores a three‐phase interface is created between carbon dioxide, water, and the membrane, and there is increased storage of carbon dioxide in nanobubbles.

### 3.3 Triphasic Gas Storage in Polymers of Intrinsic Microporosity

**Figure 6:**
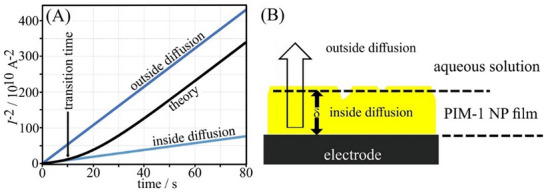
A) Cottrell plot (square of inverse current versus time) for simulated chronoamperometry data comparing the cases of i) diffusion in solution (outside diffusion), ii) diffusion in the film (inside diffusion), and iii) the theory (Equation 5) for transition between inside and outside diffusion. B) Illustration of the transition from inside diffusion to outside diffusion (with permission^[^ ^95^ ^]^).

**Figure 7:**
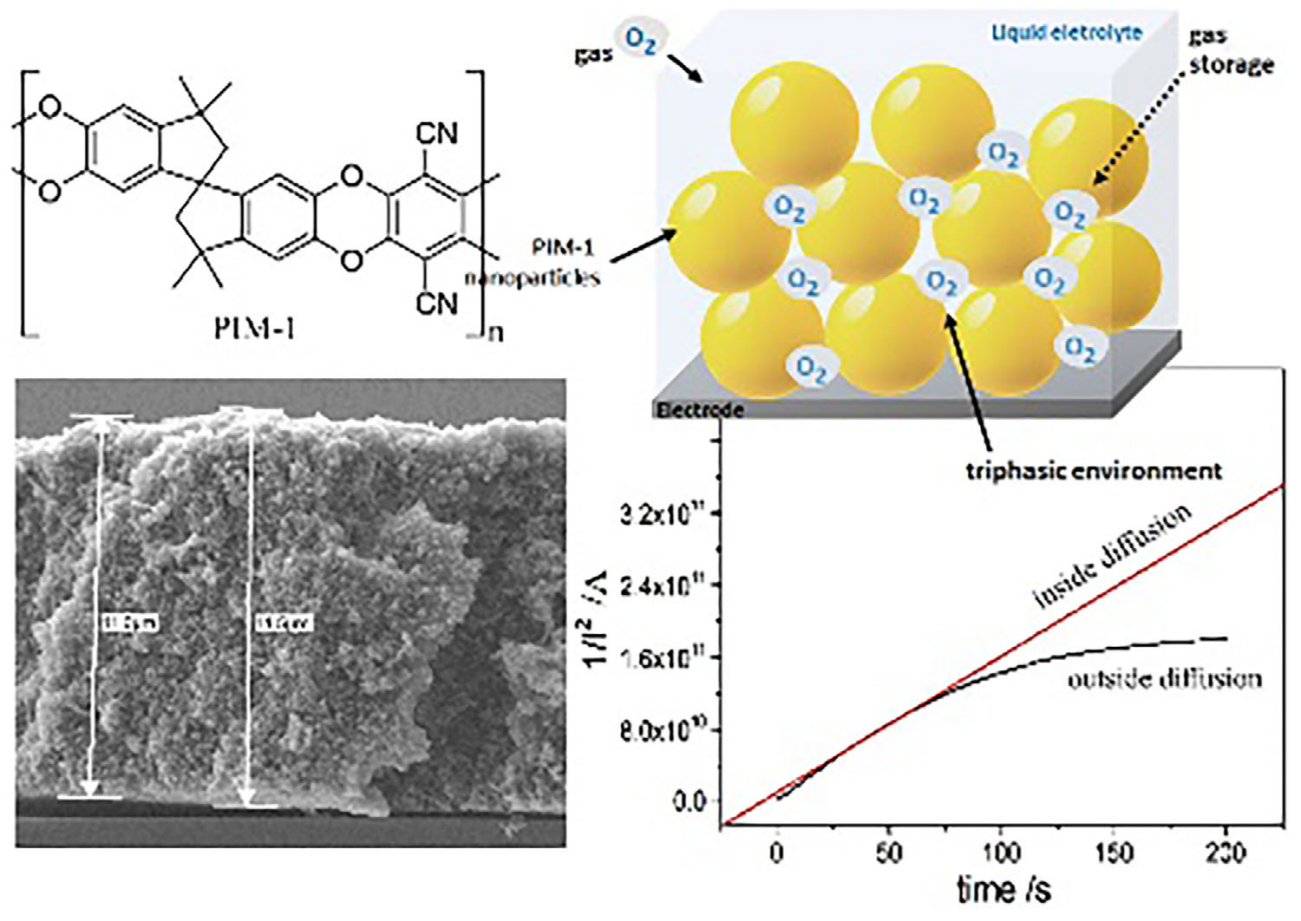
Illustration of the molecular structure of PIM‐1, the packing of nanoparticles at the electrode surface, a scanning electron micrograph of the nanoparticle film, and a Cottrell plot of transient current *I* ^−2^ versus time *t* (with permission in Ref. [11]).

The effect of PIM‐1 nanoparticle deposition as a film (**Figures** **6** and **7**) onto electrodes on apparent concentrations (*c*_app_) of oxygen and hydrogen has been investigated.^[^ ^17^ ^]^ For oxygen, it was found that the apparent concentration (*c*_app,oxygen_) increases to 50 (±5) mm in the presence of PIM‐1 nanoparticles, up from the ambient concentration, *c*_oxygen_ = 0.3 mm. Values for *c*_app_ and *D*_app_ were extracted from chronoamperometry data which show the transition from inside diffusion to outside diffusion. A simplified theoretical model was based on Cottrellian diffusion inside and outside of the microporous film (Equation 5). Introducing a film with thickness *δ* on the electrode surface, and parameter $u=K\sqrt{D_{app}/D}\left(K=c_{app}/c\right.$ is the partitioning constant for the reactant), the Cottrell equation can be rewritten based on work by Peerce and Bard.^[^ ^119^ ^]^

(5) $$I\;\left(t\right)=\frac{\mathrm{nFAc}\sqrt{D}}{\sqrt{\pi t}}\;u\left\{1+2\sum_{j=1}{\left(\frac{1-u}{1+u}\right)}^{j}exp\left(-\frac{j^{2}\delta^{2}}{Dt}\right)\right\}$$

Figure 6A shows a Cottrell plot for (i) pure inside diffusion, (ii) pure outside diffusion, and (iii) for theory data generated with Equation 5, showing the transition from inside to outside diffusion. A transition time can be estimated at the point where a switch between the two linear domains occurs. This model is still too restrictive to directly fit to experimental data, but the transition time allows to estimate *D*_app_ which then (based on the Cottrell slope for inside diffusion) gives an estimate of *c*_app_.

For the case of oxygen binding, the presence of oxygen in the triphasic environment was confirmed by cyclic voltammetry, where increasing thicknesses of the PIM‐1 deposit were correlated with an increased current peak for the reduction of O_2_. A PIM‐1 film with an approximate thickness of 12 (±2.4) µm was associated with a threefold increase in the reduction current.^[^ ^11^ ^]^ This suggested that PIM‐1 increased oxygen storage at the electrode surface by creating a triphasic environment.

Although the concentration of oxygen is increased due to triphasic gas storage, the presence of PIM‐1 decreases the apparent diffusion coefficient from 2.8 × 10^−9^ to 1.6 × 10^−12^ m^2^ s^−1^ with an ≈12 (±2.4) µm PIM‐1 layer.^[^ ^11^ ^]^ This is likely due to rigidity and the size of the micropores: many PIMs are selective for gas molecules based on size, which has led to the use of PIMs in gas separation applications: smaller molecules can be transported through pores, while larger molecules are immobilised within them.^[^ ^120^ ^]^ The relatively large O_2_ molecules do not move by pore diffusion; instead, gas transport occurs by a solution‐diffusion mechanism: the O_2_ molecules adsorb, diffuse through the network, then desorb. O_2_ molecules cannot access all the free volume due to “bottlenecks” present in the microporous network.^[^ ^121^ ^]^ It is also possible that the energy required to overcome the adsorption energy slows diffusion. More generally, PIMs are glassy polymers and therefore diffusion rates for guest molecules slow down to the level typical for rigid solids.

**Figure 8:**
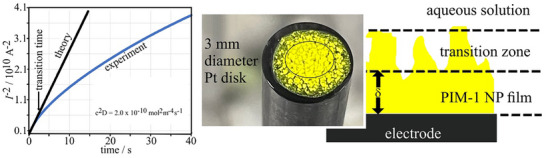
Chronoamperometry at a 3 mm diameter Pt disk electrode with 50 µg PIM‐1 immersed in 0.1 m phosphate buffer pH 7 saturated with hydrogen gas. The potential is stepped from open circuit to 0.0 V versus SCE. The theory line is based on Equation 4 (with permission^[^ ^95^ ^]^).

The apparent concentration of hydrogen at an electrode surface bearing a 12 (±6) µm PIM‐1 gas management layer (**Figure** **8** and **Table** **3**) was found to be 80 (±41) mm, compared to 0.08 (±0.04) mm on a bare electrode.^[^ ^96^ ^]^ Despite the large error, which arises from uncertainty in the film thickness due to heterogeneity of the PIM‐1 film, there is clear triphasic H_2_ storage in the layer. The diffusivity of hydrogen on a bare electrode was quantified as 5.0 × 10^−9^ m^2^ s^−1^, which decreased to 12 (±6) × 10^−12^ m^2^ s^−1^ in the PIM‐1 layer. It may be expected that the diffusivity would remain largely unchanged because the free volume of the microporous network should be accessible to the small H_2_ molecules. However, the lack of molecular backbone motion and some hydrogen adsorbing to PIM‐1 raises the activation energy of diffusivity, hence the slower diffusion. The unadsorbed hydrogen is likely trapped mainly as nanobubbles in the triphasic environment, and it may diffuse to adjacent bubbles through solution. The apparent diffusivity of H_2_ in PIM‐1 (12 (±6) × 10^−12^ m^2^ s^−1^) is still approximately ten times larger than the diffusivity of O_2_ within a PIM‐1 deposit of approximately equal thickness (1.6 × 10^−12^ m^2^ s^−1^). This supports the idea that there is more of the free volume of the microporous network available to the smaller H_2_ molecules, compared to the larger O_2_ molecules, which are restricted by bottlenecks.

**Table 3:** Summary of preliminary experimental data from hydrogen oxidation chronoamperometry at a bare i) and at modified Pt PIM‐1 nanoparticulate film electrodes ii–v) of varying thickness in phosphate buffer (pH 7.0, 0.1 m) under saturated H_2_ conditions (with permission^[^ ^96^ ^]^).

Amount of PIM‐1/µg	Approx. Transition Time/s ^a^ ^)^	Approx. Film Thickness/µm ^a^ ^)^	*c* ^2^ *D* /mol^2^ m^−4^ s^−1^	*D*_app,hydrogen_ /m^2^ s^−1^ ^a^ ^)^	*c*_app,hydrogen_ /mm ^a^ ^)^
0	‐	‐	3.3 × 10^−11^	5.0 × 10^−9^	0.08 (± 0.04)
50	5 (±2)	3 (±1)	2.0 × 10^−10^	1.8 (± 0.9) × 10^−12^	10 (± 5)
100	7 (±3)	4 (±2)	1.8 × 10^−9^	2.3 (±1.2) × 10^−12^	28 (±14)
200	20 (±8) ^b)^	7 (±3)	3.7 × 10^−9^	2.4 (±1.2) × 10^−12^	39 (±20)
400	12 (±5) ^b)^	12 (±6)	80 × 10^−9^	12 (±6) × 10^−12^	81 (±41)

^a)^ Errors estimated based on assumption of 50% uncertainty in the film thickness.

^b)^ The transition time is expected to monotonically increase but experimental error possibly due to film roughness here seems significant.

The storage of hydrogen within an aqueous suspension of PIM‐1 nanoparticles can be verified by ^1^H‐NMR spectroscopy. Figure 8 shows ^1^H‐NMR data with the water signal at 4.8 ppm suppressed. Peaks for PIM‐1 particles are not observed. The stored hydrogen (H_2_) is observed at 4.57 ppm up‐shifted slightly from the signal for gaseous hydrogen.^[^ ^122^, ^123^, ^124^, ^125^ ^]^ The amount of stored hydrogen can be estimated as typically one H_2_ per one PIM‐1 monomer unit under these conditions.

**Figure 9:**
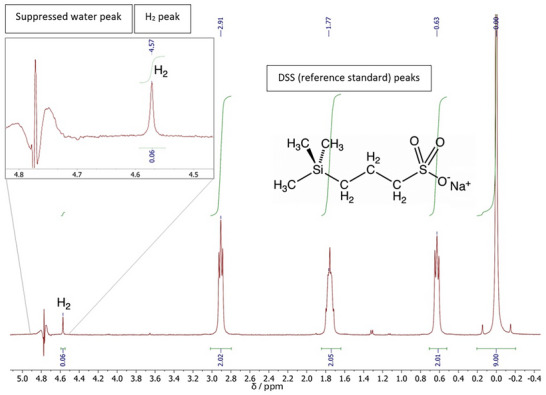
^1^H‐NMR data (400 MHz Bruker Neo spectrometer equipped with an iProbe) of H_2_ storage in PIM‐1 nanoparticles (molecular weight of the monomer 460 g mol^−1^) suspended in D_2_O. The H_2_O signal at 4.8 ppm was suppressed by pre‐saturation. Sodium trimethylsilylpropanesulfonate (DSS) was used as the reference internal standard. The H_2_ integral corresponds to ≈2 mm hydrogen (see text in Ref. [127]).

**Figure** **9** allows for accurate integration to quantify and relate H_2_ storage to amount/volume of PIM‐1. The concentration of H_2_ gas may be estimated from the integration values of the H_2_ peak at *δ* 4.57 ppm relative to the integration of the sodium trimethylsilylpropanesulfonate (DSS) peak: the integral value needs to be multiplied by 100/75 to account for NMR inactive *para*‐H_2_, which is present in the *ortho*/*para* ratio 75:25.^[^ ^126^ ^]^ From this, the concentration of H_2_ stored in PIM‐1 is found to increase of ≈25 times from the known concentration of H_2_ in aqueous phase (0.08 mm ^[^ ^96^ ^]^). This is consistent with the previously estimated concentration of triphasic H_2_ storage (80 mm in a film deposit of PIM‐1 nanoparticles). The gravimetric capacity of PIM‐1 in ambient triphasic conditions may also be estimated using the known mass of PIM‐1 in the sample, giving a value of typically 1.0 wt.%, based on PIM‐1 being notably similar to the storage capacity of dry PIM‐1 at 1 bar and cryogenic temperatures (77 K, 1.04 wt.%^[^ ^70^ ^]^).

## 4 Microporous Materials for Triphasic Processes and Interfaces II.: Catalysis

**Figure 10:**
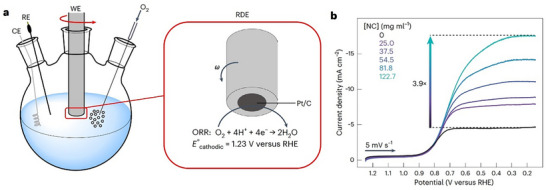
a) A schematic highlighting the rotating disk electrode (RDE) set‐up employed for the oxygen reduction reaction (ORR) catalysis experiments using a glassy carbon working electrode (WE) with a Pt/C catalyst film, a leakless Ag/AgCl‐based reference electrode (RE) and a Pt‐mesh counter electrode (CE). b) RDE voltammograms collected in O_2_‐saturated phosphate‐buffered water (0.5 m KP_i_) solutions at pH 7.0 containing 0–122.7 mg mL^−1^ of A‐silicalite‐1‐NCs at a scan rate of 5 mV s^−1^ and an electrode rotation rate of 1600 rpm (with permission in Ref. [4]).

The supply of gases to electrocatalysts is important in many types of electrode processes. Novel PTFE gas diffusion layer‐based electrodes for carbon dioxide reduction have been developed by Strasser.^[^ ^128^ ^]^ An enhanced activity for electrochemical oxygen reduction was noted in “microporous water.” Thorarinsdottir and coworkers^[^ ^4^ ^]^ have recently pointed out the effect of triphasic gas storage in silicate‐1 zeolite nanocrystals on electrochemical oxygen reactivity. Disk‐shaped nanocrystals of typically 200 nm diameter were employed to improve oxygen storage in aqueous phosphate buffer at pH 7. **Figure** **10** shows data for rotating disk voltammetry for the 4‐electron reduction of oxygen under steady state conditions (1600 rpm). With the addition of more silicate‐1 nanocrystals (NC) the current can be seen to more than triple, indicative of additional flux of oxygen (associated with the apparent concentration) from the microporous host dispersed in water.

**Figure 11:**
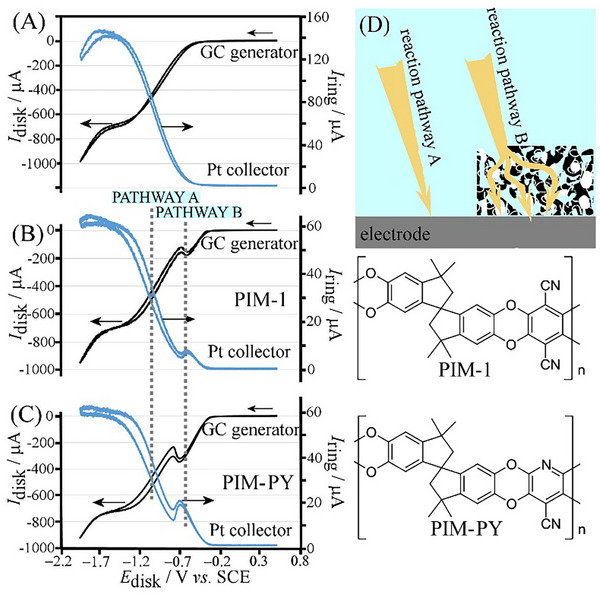
Cyclic voltammograms (scan rate 50 mV s^−1^; rotating ring‐disk electrode with a 5.5 mm diameter glassy carbon disc and a 2 mm wide platinum ring; 1500 rpm; ring potential +0.3 V vs SCE) for the reduction of oxygen (1 bar oxygen purged solution) in 0.01 m phosphate buffer solution at pH 7 for A) bare glassy carbon, B) PIM‐1 nanoparticle modified glassy carbon, and C) PIM‐PY nanoparticle modified glassy carbon. D) Schematic drawing of reaction pathway A (gas molecules diffuse from solution to the electrode surface) and reaction pathway B (gas molecules accumulate in the intrinsically microporous polymer host and react at the electrode surface with apparently higher activity) (with permission in Ref. [17]).

In contrast to the case of microporous material in solution, for PIM‐1 nanoparticles deposited onto a glassy carbon rotating disc electrode (with platinum ring), a new “catalytic” oxygen reduction current peak was observed under steady state conditions. **Figure** **11** shows both the disk current (2‐electron oxygen reduction to H_2_O_2_) and the ring current (2‐electron oxidation of H_2_O_2_). In the absence of PIM‐1 or PIM‐PY (see molecular structures in Figure 11) the oxygen reduction at the generator electrode produces H_2_O_2_, which is detected at the Pt‐ring collector electrode. When depositing PIM‐1 nanoparticles, the onset for both oxygen reduction and H_2_O_2_ detection is shifted positive by ≈0.24 V, consistent with a local increase in oxygen concentration (or partial pressure) by three orders of magnitude. Both PIM‐1 and PIM‐PY nanoparticles result in very similar effects. When scanning the generator potential more negative, the effect (indicated as reaction pathway B) is outrun by the conventional process (reaction pathway A).

**Figure 12:**
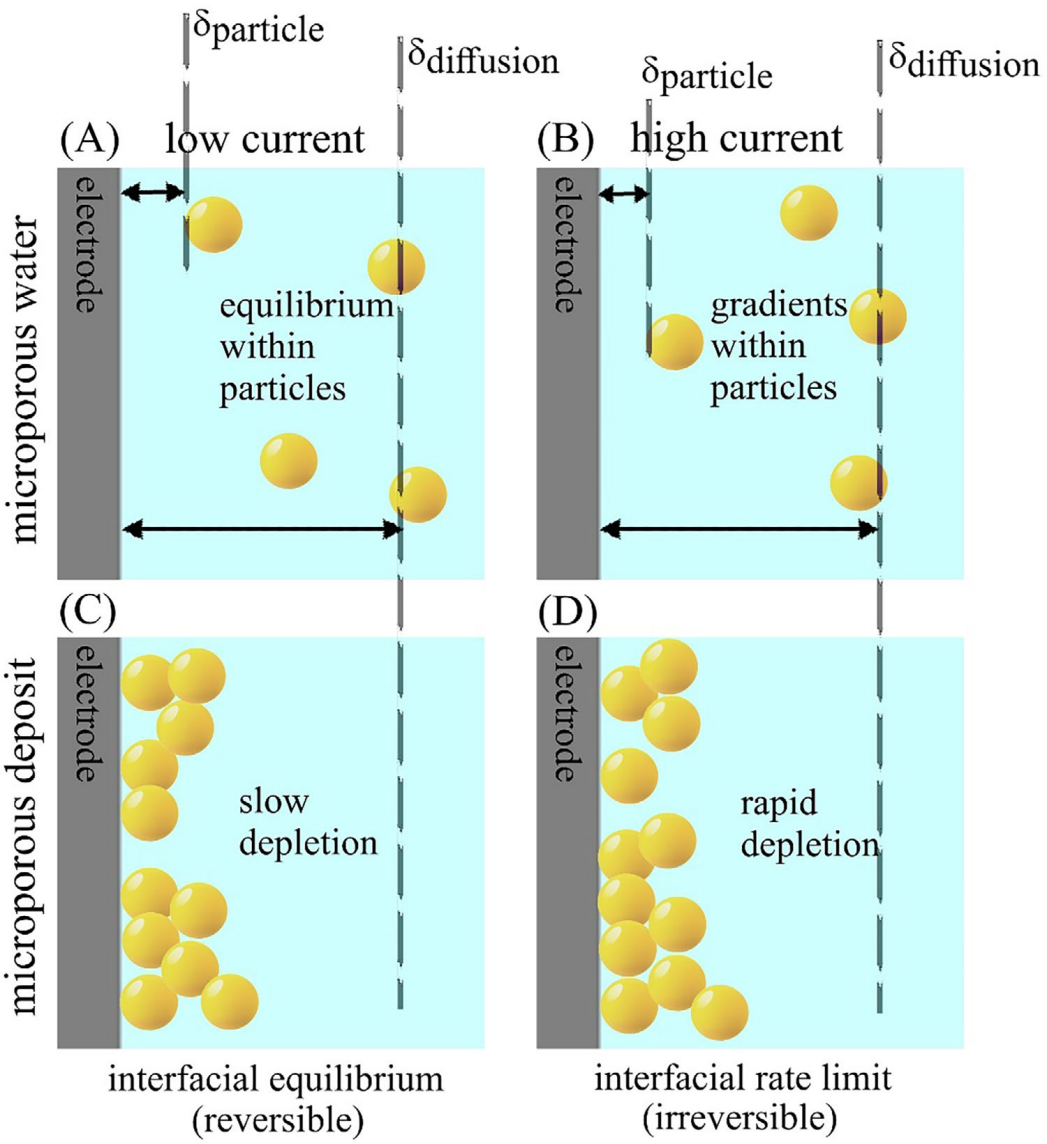
Illustration of the kinetic limiting cases for A,B) microporous water and C,D) microporous deposits assuming low current (A,C;, i.e., low gas flux and “reversible” conditions at the particle|solution interface) or high current (B,D;, i.e., high gas flux and “irreversible” conditions at the particle|solution interface).

For both (I) particles suspended or “microporous water” and (II) particles deposited directly onto the electrode surface, similar enhancement mechanisms are likely. However, the transport conditions and location of the particles may affect the details of gas release. This is illustrated schematically in **Figure** **12** for microporous water (A, B) and microporous deposits (C, D).

The diffusion layer δ_diffusion_ is time‐dependent and agitation dependent. For long times and/or weak agitation, the current (i.e., gas flux) is low. Gas stored in particles can equilibrate at the particle|solution interface (reversible interfacial conditions), and the increase of the apparent concentration *c*_app_ can be observed, for example, by voltammetry. Depletion of gas within particles may occur as they approach the electrode. The concentration difference and the gap δ_particle_ define the transport of gaseous species from particle to electrode surface. At short times or for stronger agitation, the current is high and the equilibrium at the particle|solution interface is disturbed (irreversible interfacial conditions). Both a slow transfer of the gaseous species or, perhaps more likely, slow transport within the particle could play a role.

**Figure 13:**
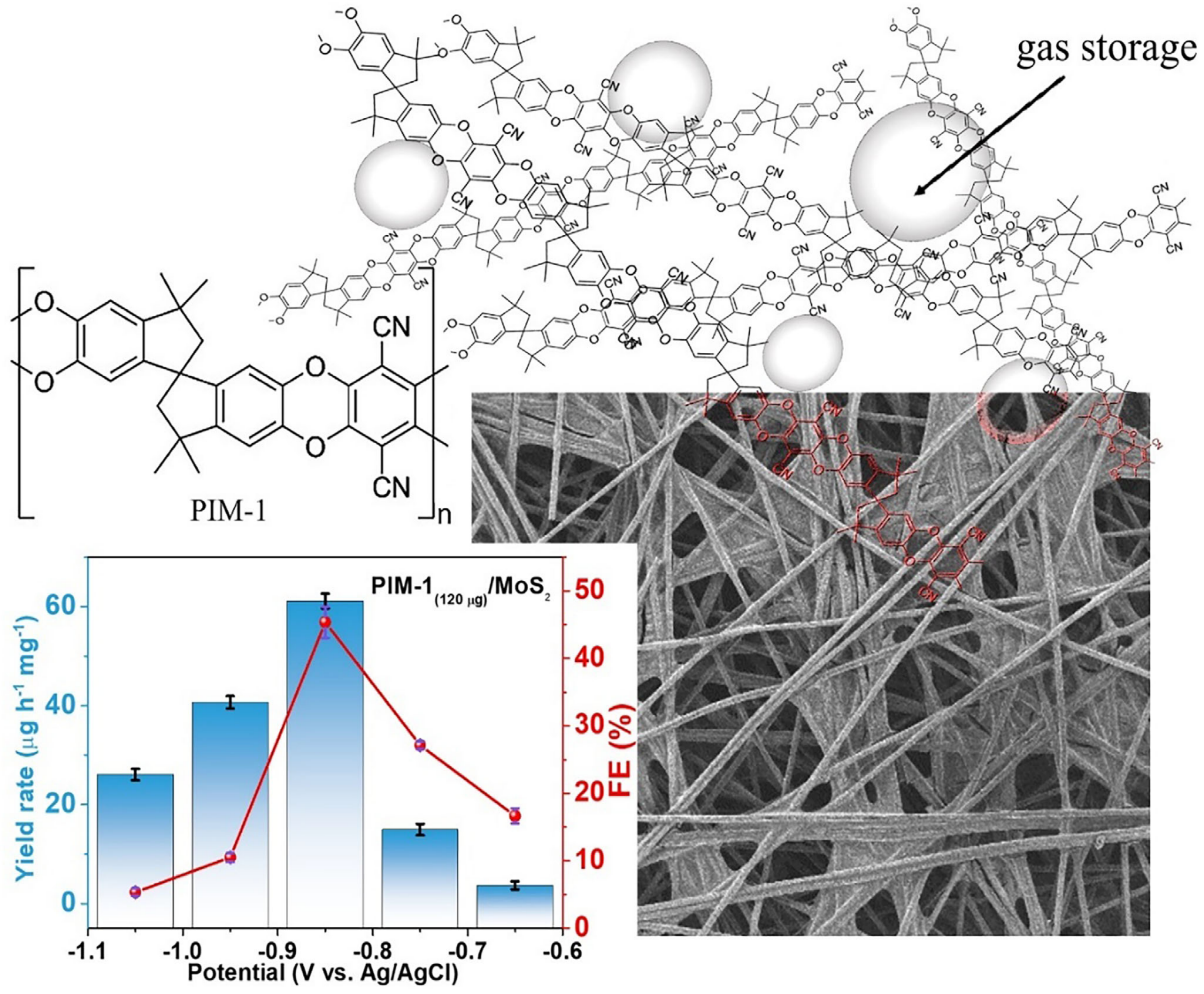
Illustration of gas storage in PIM‐1, molecular structure of PIM‐1, electron micrograph of carbon paper substrate with amorphous MoS_2_ and PIM‐1 deposit, and a plot of ammonia production as a function of applied potential with/without PMI‐1 for chronoamperometry electrolysis (2 h) at various potentials for PIM‐1(120 µg)/MoS_2_/CP and NH_3_ yield rate and faradaic efficiency (FE) values for PIM‐1(120 µg)/MoS_2_/CP. (with permission^[^ ^130^ ^]^).

Similar to the effects observed for oxygen reduction, the presence of microporous PIM‐1 affects the electrocatalytic reduction of nitrogen, N_2_, to ammonia, NH_3_. The electrocatalytic reduction of nitrogen on electrodeposited amorphous MoS_2_ was investigated by Almeida and co‐workers.^[^ ^129^ ^]^ When applying PIM‐1 nanoparticles to this catalyst, both oxygen reduction and nitrogen reduction were enhanced.^[^ ^130^ ^]^ **Figure** **13** shows a scanning electron micrograph of the carbon paper substrate with both amorphous MoS_2_ and PIM‐1 nanoparticles deposited. The plot summarises the NH_3_ production as a function of the applied potential, and in the presence and absence of PIM‐1. The microporous polymer appears to approximately double the NH_3_ production at all potentials, with a maximum in performance at −0.85 V vs Ag/AgCl.

**Figure 14:**
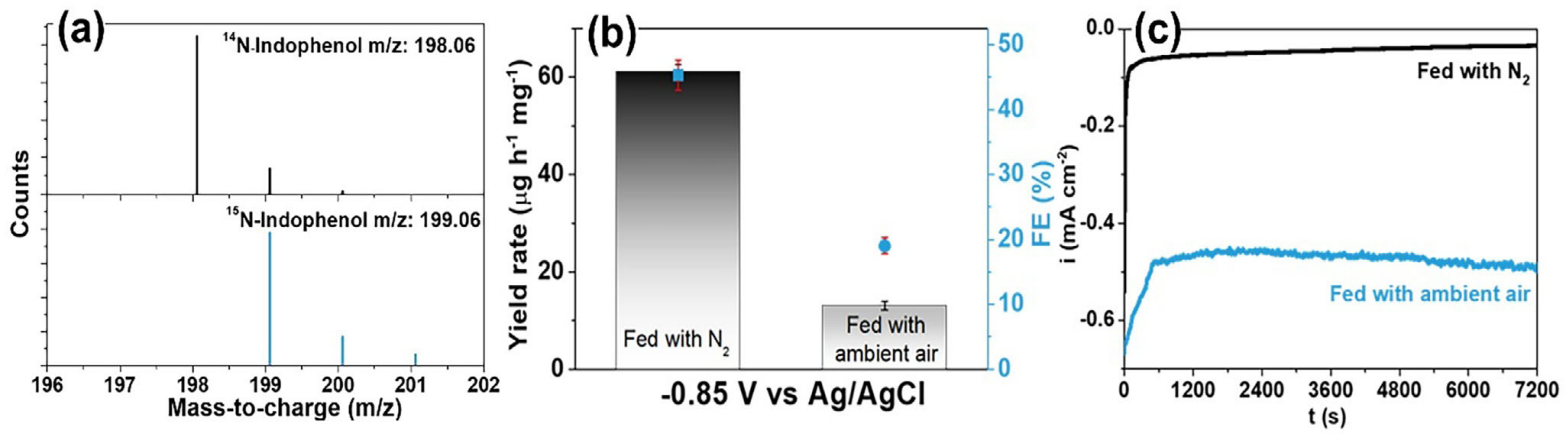
a) The mass spectrum for electrolyte samples collected after isotope labelling nitrogen reduction experiments by PIM‐1(120 µg)/MoS_2_/CP catalyst, demonstrating formation of ^14^N‐indophenol and ^15^N‐indophenol, respectively. b) Comparison of NH_3_ yield rate and faradaic efficiency values for PIM‐1(120 µg)/MoS_2_/CP in N_2_‐saturated and air‐saturated solution. c) Chronoamperometry electrolysis (2 h) at −0.85 V vs Ag/AgCl in N_2_‐saturated (black curve) and air‐saturated (blue curve) solution for PIM‐1(120 µg)/MoS_2_/CP (with permission in Ref. [130]).

The process was investigated for the reduction of ^15^N_2_ to give isotopically labelled ammonia, which was then converted to indophenol and detected by mass spectroscopy (**Figure** **14**a). Perhaps surprisingly, the gas feed could be replaced with a direct air feed (79% nitrogen), and the production of ammonia seemed possible even in the presence of ambient oxygen, although at much lower efficiency (Figure 14b,c). This process can be compared to those in natural nitrogenases. The local PIM‐1 deposit may be acting similarly to “gas tunnel” structures in natural enzymes.

Localised triphasic gas storage at the electrode surface could be useful for a wider range of electrocatalytic processes and materials that allow gas storage in wet conditions. For example, PIMs or MOFs could be integrated into electrode systems (e.g., gas diffusion electrodes) to affect reaction rates and pathways and to mimic biological redox catalysis.

## 5 Conclusion and Outlook

Microporous materials with dry (biphasic) and wet (triphasic) gas storage effects have been discussed in the context of electrochemical processes. Triphasic gas storage in aqueous media has been shown to increase the apparent concentration of gases, overcoming the challenge of gas solubility in aqueous media and enhancing electrochemical reactions involving gases. Some microporous materials, such as polymers of intrinsic microporosity (PIMs) and metal–organic frameworks (MOFs), exhibit some of the desirable properties for triphasic gas storage, namely high surface areas, high microporosity, tuneable properties such as hydrophobicity, chemical, thermal, and mechanical stability in aqueous media, and processability. Better measurements will be necessary (e.g., maybe based on nanoparticle impact current transients^[^ ^131^, ^132^ ^]^) to unravel effects from *c*_app_ and *D*_app_ for different materials. Better theoretical models describing the gas uptake and release could be developed in conjunction with chronoamperometric data from experiments.

The challenge of designing materials that are effective in all these areas may involve functionalisation of microporous materials, heat treatments, alternative synthesis methods, and forming of composites. The dry gas storage of microporous materials, while well understood, is not yet scalable to a hydrogen economy and is largely performed at cryogenic temperatures. Triphasic gas storage in microporous materials is less understood, and more research is needed into quantifying how much gas is stored (e.g., by electrochemical, thermogravimetric, or spectroscopic experimental methods). For example, we showed that NMR can be used to quantify stored H_2_ within PIM‐1 particles suspended in water, which supported the conclusions of triphasic gas storage derived from electrochemical reactions.

There is scope for effective triphasic gas storage within microporous materials without the need for high pressure or cryogenic conditions. The limits of triphasic gas storage are currently unknown, but enhanced gas storage by a three orders of magnitude apparent concentration increase suggests that gas storage in batteries (rechargeable batteries or redox flow batteries) could be feasible. The concept of triphasic storage may have wider use in electrochemistry, and more specifically in sensing, electrocatalysis, electrosynthesis, and green energy storage and production (fuel cells). Crucially, more microporous materials for triphasic gas storage need to be developed and investigated, including bio‐related materials (e.g., based on modified proteins) to overcome challenges in costs and sustainability.

## Conflict of Interest

The authors declare no conflict of interest.
